# Improving Pneumococcal Vaccination Coverage Among Older Adults in a Rural Hospital: A Plan-Do-Study-Act (PDSA)-Based Quality Improvement Project

**DOI:** 10.7759/cureus.97353

**Published:** 2025-11-20

**Authors:** Ryuichi Ohta, Koki Kato, Chiaki Sano

**Affiliations:** 1 Community Care, Unnan City Hospital, Unnan, JPN; 2 Family Medicine, Madoka Family Clinic, Ogori, JPN; 3 Community Medicine Management, Shimane University Faculty of Medicine, Izumo, JPN

**Keywords:** family medicine, general medicine, older adults, pneumococcal vaccines, quality improvement, rural, rural health services, vaccination coverage

## Abstract

Background

Pneumococcal pneumonia remains a leading cause of morbidity and mortality among older adults, particularly in rural areas with limited healthcare access. Despite national recommendations, vaccination uptake in rural Japan remains suboptimal. Evidence suggests that patient education, reminder systems, and streamlined workflows can enhance preventive care in outpatient settings. This quality improvement (QI) project aimed to increase pneumococcal vaccination rates by at least 20% within three months among older patients in a rural Japanese hospital.

Methods

The project was conducted at Unnan City Hospital, a rural healthcare facility with a high proportion of older adults, from February to April 2025. Using the Plan-Do-Study-Act (PDSA) framework, three low-cost interventions were implemented: (1) patient and staff educational brochures; (2) electronic medical record (EMR)-based reminders; and (3) a standardized vaccination flowsheet. Eligible patients were aged ≥65 years with chronic conditions who regularly attended outpatient visits. Outcome measures included weekly vaccination rates; process measures included recommendation and reminder rates; and balance measures included staff satisfaction and perceived workload. We used run charts and interrupted time-series analysis (ITSA) to assess temporal changes; this method allows for the analysis of trends over time but has a limited ability to determine causality.

Results

Baseline vaccination coverage averaged 17.4% over eight months prior to the intervention. Following the QI project, vaccination rates increased to 65.0% within three months. ITSA demonstrated a significant post-intervention increase of 3.97 percentage points per week (p < 0.001). Physician recommendation rates improved from 24% to 83%, and reminder rates consistently exceeded 91%. Staff satisfaction with workflow and team communication improved, while perceived workload remained manageable. Qualitative feedback informed iterative adjustments, including daily vaccination limits and enhanced communication.

Conclusions

This multicomponent intervention was associated with substantial increases in pneumococcal vaccination rates among older adults in a rural hospital. Integrating EMR-based reminders, patient education, and interprofessional collaboration into routine practice proved feasible and scalable. This model may serve as a practical framework for enhancing adult vaccination in similar rural healthcare settings.

## Introduction

Pneumococcal pneumonia poses a critical health threat to older patients, significantly impacting their quality of life and contributing to higher morbidity and mortality rates [1,2]. This condition, caused by *Streptococcus pneumoniae*, can lead to severe complications in older adults, whose immune systems are often weakened due to age and comorbidities [3]. Consequently, effective measures to prevent pneumococcal pneumonia are essential to safeguarding the health and well-being of this vulnerable population [3].

Vaccination against *S. pneumoniae* is a practical and proven strategy for preventing pneumococcal pneumonia [4-6]. High vaccination rates have been shown to reduce mortality among older patients, subsequently enhancing their quality of life following treatment [5,6]. However, despite strong national and international recommendations, pneumococcal vaccination uptake remains suboptimal, particularly in rural areas where healthcare access and continuity are limited [7]. Several studies have identified common barriers to uptake, including limited awareness or understanding of the vaccine’s benefits, concerns about side effects, cost-related hesitations, and lack of strong recommendations from healthcare providers [8-10]. Additionally, system-level issues such as the absence of structured reminder systems or standardized workflows further contribute to underutilization [11,12]. System-level issues, such as inconsistent reminder systems and fragmented workflows, further contribute to low uptake in primary care settings.

Pneumococcal vaccination is a cost-effective vaccine to prevent invasive pneumococcal infections. This vaccine is provided once every five years, and the raw cost is not expensive: $80 (approximately $16 per year), which is reduced to $30 (approximately $6 per year) by the financial support of the local government. However, this governmental subsidy is typically limited to individuals receiving their first pneumococcal vaccination at age 65. Compared with influenza and COVID-19 vaccination, which cost approximately $40 per year, this vaccine is recommended for 65-year-old patients by the Japanese government.

In our hospital, pneumococcal vaccination coverage among eligible older adults averaged only 17.4% over the previous eight months, far below national expectations and highlighting a substantial local care gap. This low uptake suggests not only patient-level hesitations but also systemic determinants such as the absence of standardized physician reminders, variability in recommendation practices, and unclear workflows for vaccination. These locally observed challenges reflect broader patterns described in the literature and underscore the need for structured quality improvement (QI) efforts.

Previous interventions suggest that multicomponent strategies, including patient education and provider reminders, can increase vaccination uptake [11-15]. Our earlier meta-analysis demonstrated that such interventions were generally effective across diverse healthcare settings, though implementation intensity varied [16]. To maintain focus on the QI rationale, we condensed this discussion to emphasize that simple, low-cost strategies have strong evidence and are feasible in rural outpatient contexts.

Targeted projects are necessary to address the low vaccination rates among older adults, especially in rural areas. Improving pneumococcal vaccination rates among older adults, particularly in rural areas, is crucial due to their heightened risk resulting from immune decline and comorbidities. The Japanese Ministry of Health, Labor, and Welfare, along with global guidelines such as those from the National Institute for Health and Care Excellence (NICE) and the Centers for Disease Control and Prevention (CDC), underscore pneumococcal vaccination as an essential preventive measure for adults aged 65 and above [10]. Vaccination reduces hospitalizations and mortality [1,5]. Yet uptake remains suboptimal in rural settings. Shared decision-making and culturally sensitive communication can improve acceptance [17].

Given the 17.4% baseline coverage in our hospital and the system-level barriers identified, an approach capable of iterative testing, rapid adjustment, and frontline staff engagement was required. The Plan-Do-Study-Act (PDSA) framework aligns closely with these needs, enabling targeted changes, such as implementing reminder systems, patient education, and workflow standardization, to be refined through sequential cycles. Therefore, this QI project aimed to increase pneumococcal vaccination rates by addressing modifiable workflow gaps through a structured PDSA-based approach.

## Materials and methods

Purpose

The primary objective was to increase pneumococcal vaccination uptake among older patients in a rural hospital setting by an absolute 20 percentage points (from 17.4% to ≥37.4%) within three months (February to April 2025). Secondary objectives included improving recommendation and reminder processes and evaluating staff satisfaction and workload. An a priori significance level of α = 0.05 was applied for all statistical analyses, including the interrupted time-series analysis (ITSA).

Design

This project used the PDSA cycle for QI to enhance pneumococcal vaccination rates among older adults in a rural hospital. The QI process followed the steps of identifying the problem, developing and implementing changes, and evaluating outcomes [18]. All patients were identified through a consecutive review of electronic medical records (EMRs), ensuring that every eligible individual was included without sampling or convenience-based selection. Eligibility criteria were (1) age ≥65 years and (2) at least one outpatient visit within the previous three months. Patients were excluded only if (1) records indicated contraindications to vaccination or if (2) they did not have an outpatient visit during the observation window.

The project was conducted in the outpatient department of Unnan City Hospital, a rural healthcare facility in Japan with a high proportion of elderly patients. At baseline, vaccination was provided at the discretion of physicians, with no standardized method for identifying eligible patients or issuing reminders, resulting in low vaccination coverage. The project targeted patients aged 65 and older with chronic conditions (e.g., hypertension, diabetes, and heart disease) who had at least one outpatient visit in the previous three months. Pre-change data were collected between June 1, 2024 and January 31, 2025.

Setting

Unnan City, located in southeastern Shimane Prefecture, is one of Japan’s most rural areas. In 2020, its population was 37,638 (18,145 males and 19,492 females), with 39% aged over 65, a figure projected to reach 50% by 2050 [19]. The city has 16 clinics, 12 visiting care services, three visiting nurse stations, and one public hospital: Unnan City Hospital. The hospital employs 27 physicians, 197 nurses, and various allied health professionals. The QI project was conducted by a multidisciplinary team of 11 outpatient staff: one family physician, five nurses, and five medical clerks.

Stakeholder analysis

**Figure 1:**
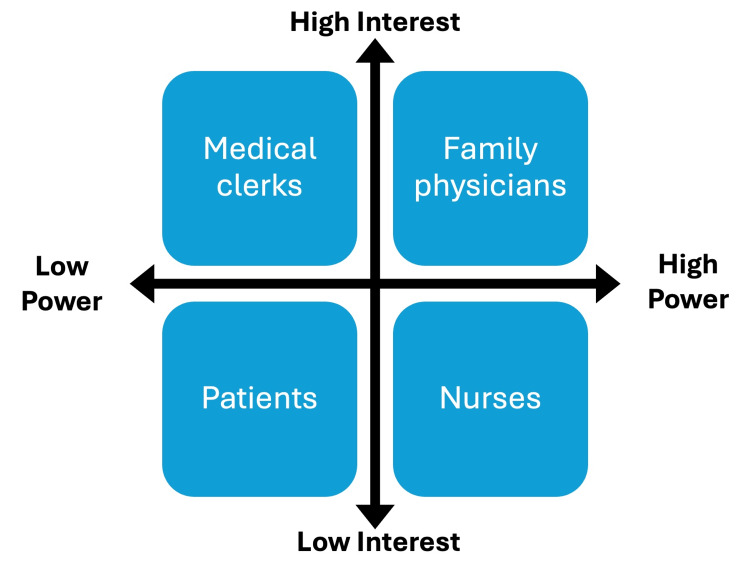
Stakeholder analysis based on the power and the interest in the project

A stakeholder matrix was developed by the QI project leader in collaboration with the core QI team, including family physicians, nurses, and medical clerks. The analysis aimed to outline the roles, responsibilities, and engagement levels of all participants involved in the QI stream to optimize collaboration and impact (Figure 1).

To tailor communication and involvement accordingly, stakeholders were analyzed using a power-interest grid, categorizing them based on their level of influence over the project and their interest in its success.

Process map of recommendations for pneumococcal vaccination for an older patient

**Figure 2:**
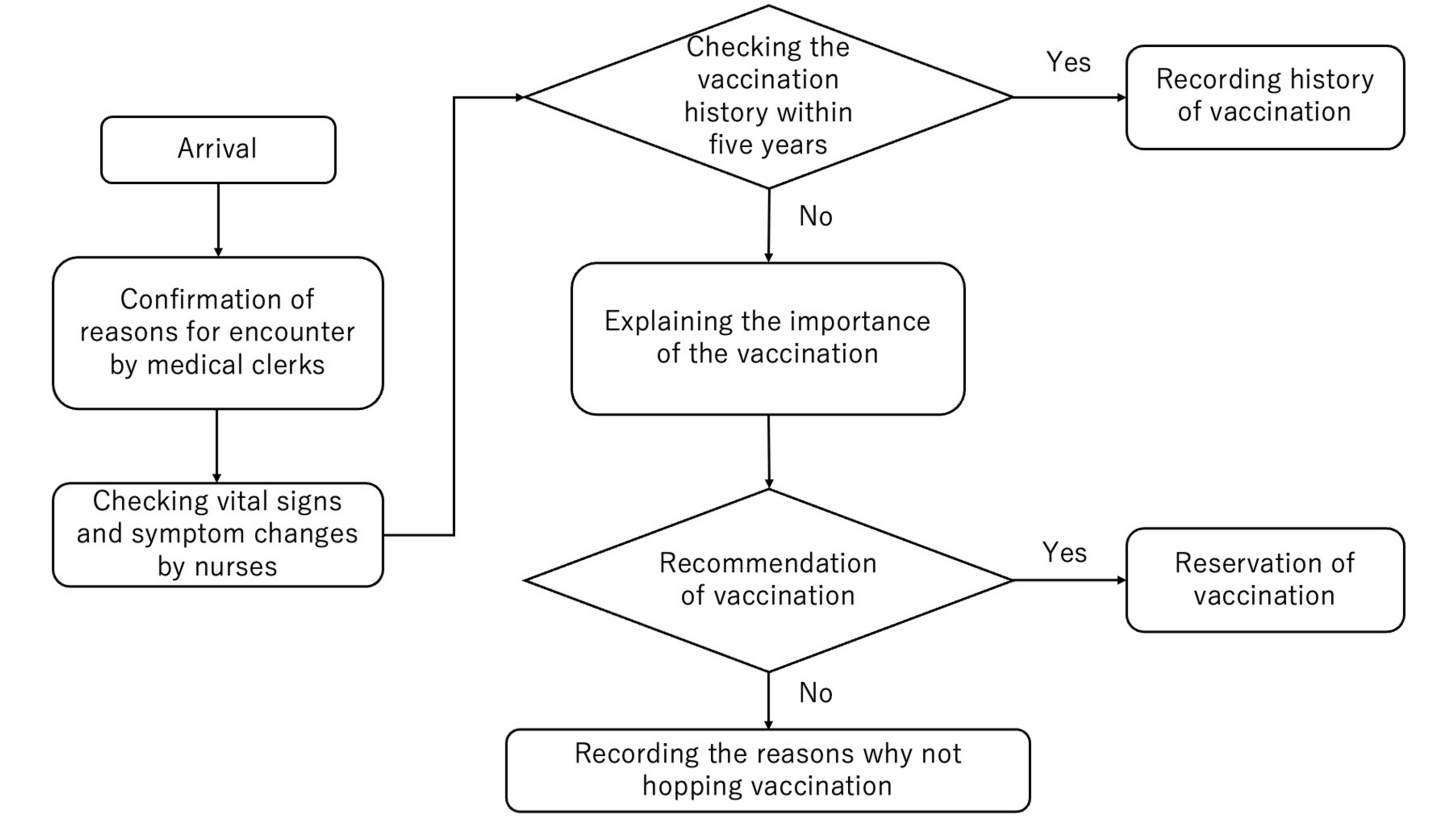
Process map of recommendations for pneumococcal vaccination for an older patient This figure was used as a flowsheet of this QI project. QI, quality improvement

The process map described the recommendation process for a pneumococcal vaccine for older patients. It was developed through informal staff interviews and direct observation of daily clinical workflows. From this process map, the vaccination frequency depended on each physician’s initiation of the recommendation of vaccination, followed by the actual vaccination process of other medical professionals. Facilitating the physician’s initiation of the recommendation was critical to QI (Figure 2).

Identification of the problem based on root cause analysis

**Figure 3:**
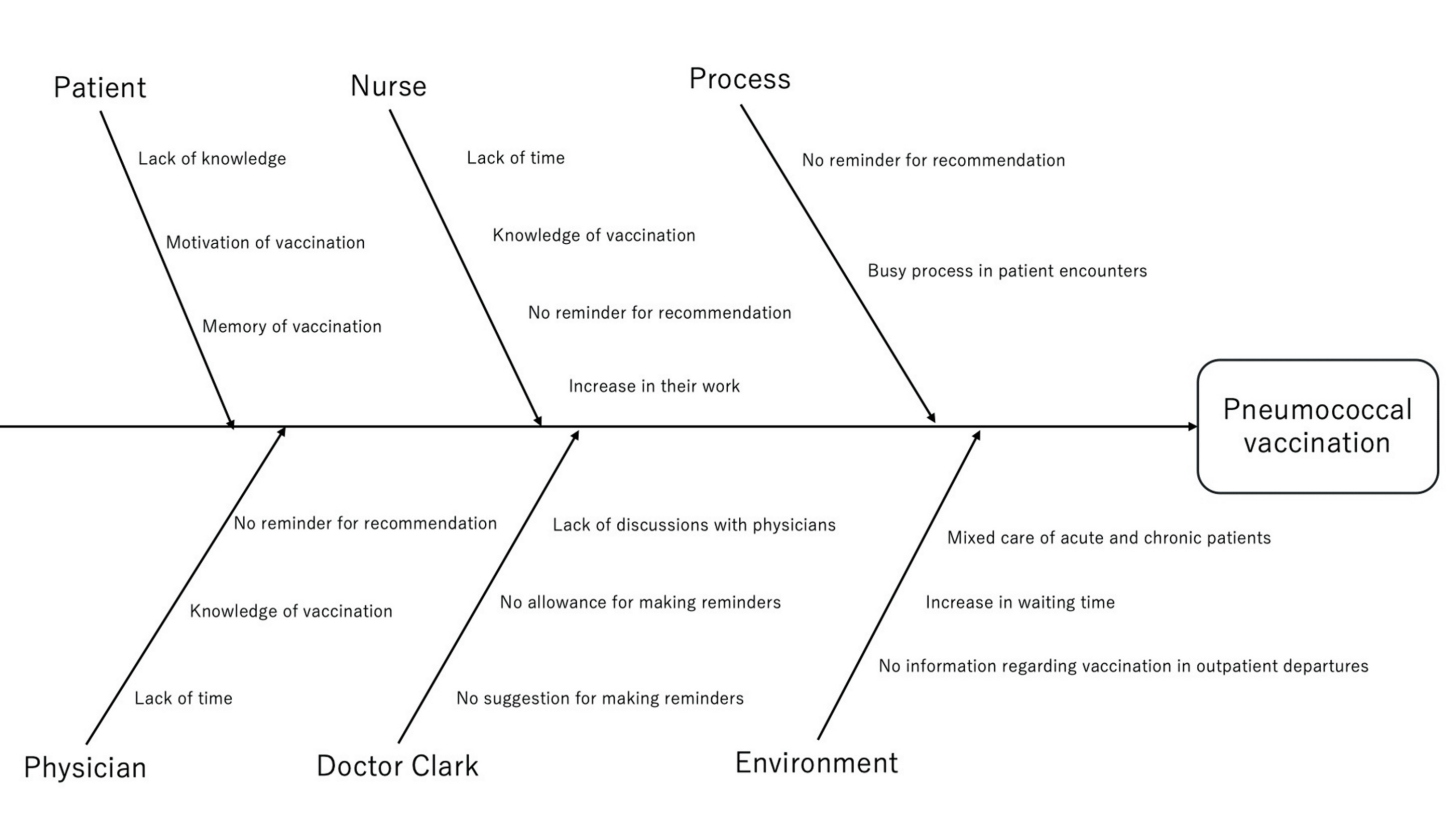
Fishbone diagram analyzing the cause and effect of pneumococcal vaccination

Fishbone diagram analysis identified potential limiting factors to understand issues to improve the promotion of pneumococcal vaccination in the hospital (Figure 3).

The diagram was developed based on informal interviews with physicians, nurses, and administrative staff, combined with direct observation of clinical workflows. Key modifiable factors included limited knowledge of the vaccine’s importance among medical professionals, insufficient time allocated for interdisciplinary discussion, and the absence of a systematic reminder mechanism. Addressing these barriers was considered essential for enhancing the frequency and consistency of vaccination recommendations.

Proposed changes

The following concrete changes were implemented following the protocol of the QI project, including scientific evidence of the changes:

Change 1: Brochures Regarding the Importance of Pneumococcal Vaccination

Brochures were provided to patients, their families, family physicians, medical clerks, and nurses to inform them about the importance of pneumococcal vaccination for older patients. This change is supported by the study of Jacobson et al. (1999), which shows that patient education materials such as brochures significantly improved vaccine uptake by increasing awareness and engagement, especially when combined with provider reinforcement [15].

Change 2: Reminder Systems

Medical clerks wrote the vaccination needs of each patient at the top of the EMRs, allowing physicians to check the reminder before each encounter. Alerts appeared prominently in the EMR, ensuring that vaccination needs were visible during consultations. Medical clerks also confirmed whether patients had read the reminder during each encounter and informed family physicians if the patient had not yet received pneumococcal vaccination.

This change is supported by studies such as Dexter et al. (2001) and Ho et al. (2019), which demonstrate that reminder systems embedded in EMRs significantly improve the delivery of preventive care, including adult vaccination uptake, by prompting consistent action from healthcare providers during clinical workflows [11,14].

Change 3: Making a Flowsheet From Recommendation to Vaccination

A flowsheet guiding the process from vaccine recommendation to administration in the outpatient department was developed by the QI project lead in collaboration with nurses and medical clerks. This was intended to facilitate vaccination and reduce the burden on QI team members by providing repeated explanations and operational guidance (Figure 2). This change is supported by See (2023), who highlights that standardized workflows and visual process maps help streamline immunization delivery by reducing practice variation, improving staff communication, and clarifying roles and responsibilities (Figure 2) [6].

Administration of the QI Team

To facilitate and drive the QI project, we conducted weekly meetings, delivered quarterly presentations summarizing project progress, and provided ongoing training for the QI team, including family physicians, nurses, and medical clerks.

Measures

Outcome Measure

The primary outcome was the vaccination rate for pneumococcal pneumonia among patients aged 65 and older who visited the outpatient section of the department of family medicine for regular follow-up of chronic diseases (Table 1).

**Table 1:** Contents of the data collection EMR, electronic medical record

Measures	Contents	Measurement methods
Outcome	Vaccination rate for pneumococcal pneumonia	The number of vaccinated patients was divided by the number of eligible candidates for pneumococcal vaccination, defined as patients aged 65 and older who regularly visited the outpatient department and had either never received the vaccine or had not received it within the past five years.
Process	Brochure supply and demand	Brochure demand was the number of eligible patients needing vaccine information, while brochure supply referred to brochures restocked weekly by clerks. Distribution and restocking data were compiled weekly.
	Physicians’ recommendation rates	The number of patients who were suggested for pneumococcal vaccination is divided by the number of candidates for pneumococcal vaccination among patients over 65 years old who regularly visit the outpatient department monthly.
	Weekly reminder rate	The number of patients who were reminded on each patient’s medical record for the vaccination, divided by the number of candidates for pneumococcal vaccination among over 65-year-old patients who regularly visit the outpatient department monthly.
Balance	Staff satisfaction and burden	Questionnaires (5-point Likert scale; 1 = very low, 5 = very high): (1) Project engagement: their overall involvement and motivation; (2) Workflow satisfaction: how the change affected daily clinical routines; (3) Usefulness of educational brochures: perceived value of brochures in patient education; (4) Clarity of the vaccination flowsheet: ease of use and helpfulness in guiding practice; (5) Effectiveness of the EMR-based reminder system: how well the reminders supported vaccine recommendations; and (6) Additional workload: how much extra effort the change requires.

Medical clerks checked medical records and detected vaccinated patients each month. This was calculated weekly as the proportion of eligible patients who received the vaccine. Eligible patients included those aged 65 and older who had either never received a pneumococcal vaccine or had received it more than five years ago. Therefore, the denominator consisted of patients over 65 who were due for their first or subsequent pneumococcal vaccination.

Process Measure

For brochure-related process measures, brochure demand was defined as the number of eligible patients aged 65 and older attending the outpatient department each week who were due to receive vaccination information. Brochure supply refers to the number of brochures that medical clerks restock and make available to staff every week. Data on distribution and restocking were logged by clerks and compiled on a weekly basis. Based on the medical records, we calculated the weekly proportion of eligible patients for whom pneumococcal vaccination was recommended by physicians (Recommendation rate) (Table 1). In addition, the reminder rate was defined as the proportion of eligible patient encounters during which medical clerks documented the need for pneumococcal vaccination in the EMR before the consultation.

Balance Measure

**Figure 4:**
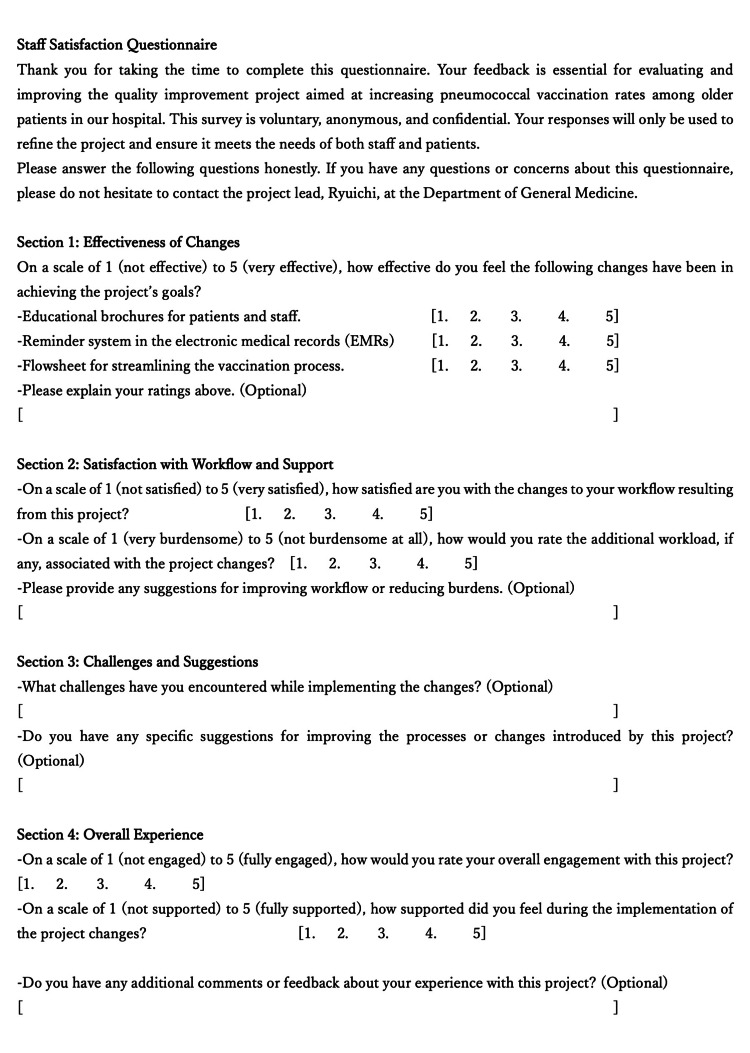
Staff Satisfaction Questionnaire Credit: Ryuichi Ohta

We evaluated the satisfaction and burden of team members. We collected feedback from team members on this QI project through questionnaires to facilitate their work in the department (Table 1). The questionnaire included items on perceived usefulness of the intervention, clarity of the workflow, ease of use of the EMR reminders, communication effectiveness, and overall workload. Staff satisfaction and burden were assessed using an anonymous six-item questionnaire administered at the start and near the end of the project. They were asked to rate on a 5-point Likert scale (1 = very low, 5 = very high) (Figure 4).

An open-ended comment section was also included for qualitative feedback. The questionnaire used in this project was developed de novo by the corresponding author (RO) for internal evaluation of staff satisfaction and workload. No copyrighted or previously published instruments were used.

Data collection

Baseline Data

To establish a baseline for evaluating the impact of the QI project, we collected data on patient demographics (age and sex), presence of chronic diseases, and pneumococcal vaccination status as outcome measures. Medical clerks retrospectively reviewed EMRs to identify the number of eligible patients who received the pneumococcal vaccination and to calculate the monthly vaccination rate. The data collection period covered eight months, from June 2024 to January 2025, encompassing pre- and post-change phases to allow for comparing trends over time and assessing the project’s impact on clinical practice.

Outcome Measure

Pneumococcal vaccination status and the date of the last vaccination were collected from the EMR, and the number of patients who were vaccinated after the change package implementation was counted weekly. The medical clerks collected these data weekly and calculated the pneumococcal vaccination rate among eligible patients (total vaccinated/total eligible patients).

Process Measure

Brochure supply and demand: Medical clerks tracked weekly brochure distribution, along with periodic inventory counts. Aggregated data were logged in a shared document accessible to project leaders.

Vaccination recommendation and reminder: Medical clerks monitored the documentation of physician recommendations for pneumococcal vaccination during the visit and the presence of vaccination reminders in the EMR. Weekly recommendation rate and weekly reminder rate were calculated.

Balance Measure

For the balance measures, data collection focused on assessing the impact of the changes on staff satisfaction, workload, and operational efficiency. Regarding staff satisfaction and feedback, anonymous questionnaires were collected twice after the first and second cycles of change implementation to monitor their satisfaction with the change process, perceived effectiveness of the changes, how much time they needed to work additionally for this QI project, and suggestions for improvement or challenges faced in implementation (Figure 4).

Implementation of changes

**Figure 5:**
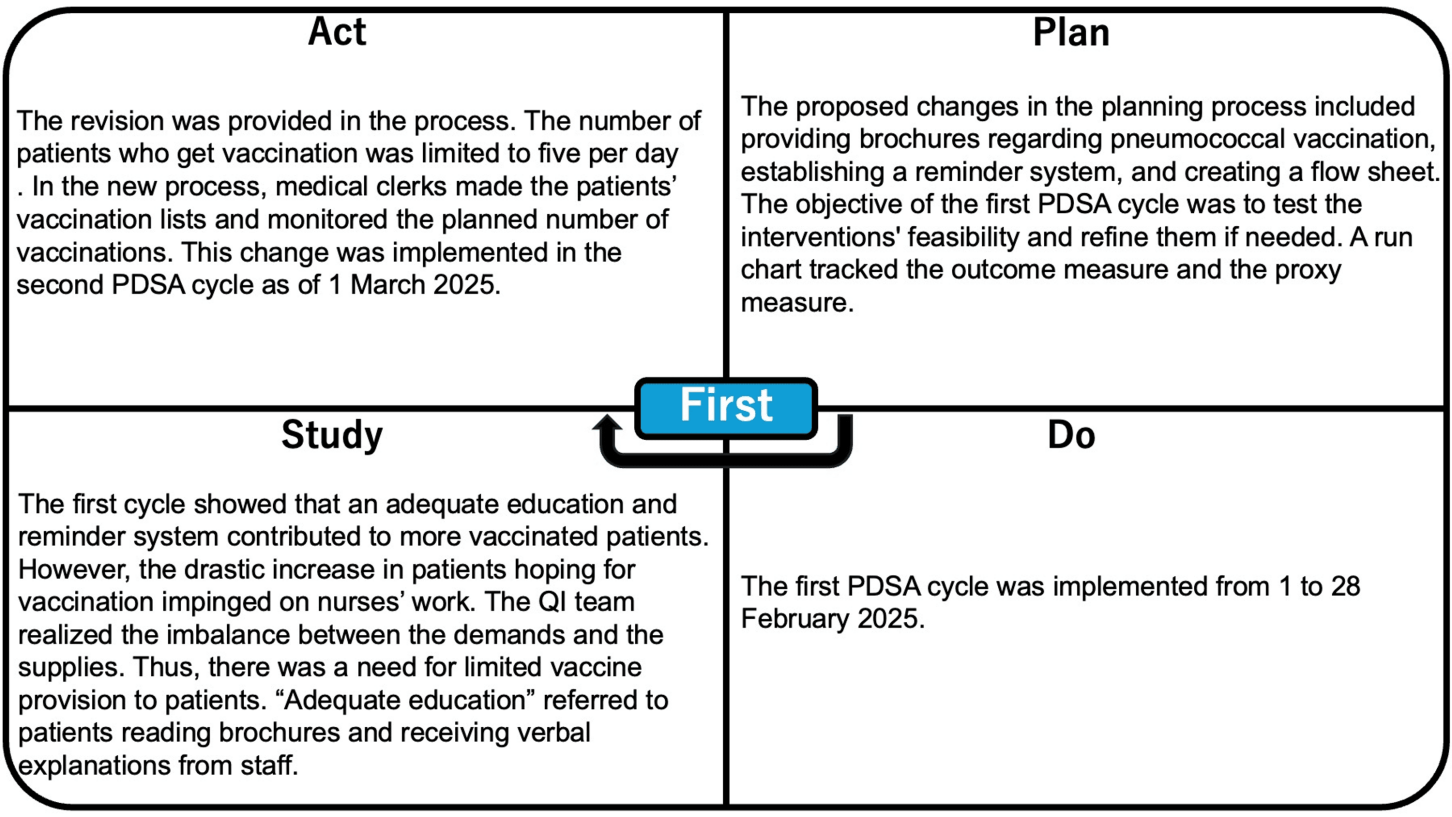
Process of the first PDSA cycle PDSA, Plan-Do-Study-Act; QI, quality improvement

**Figure 6:**
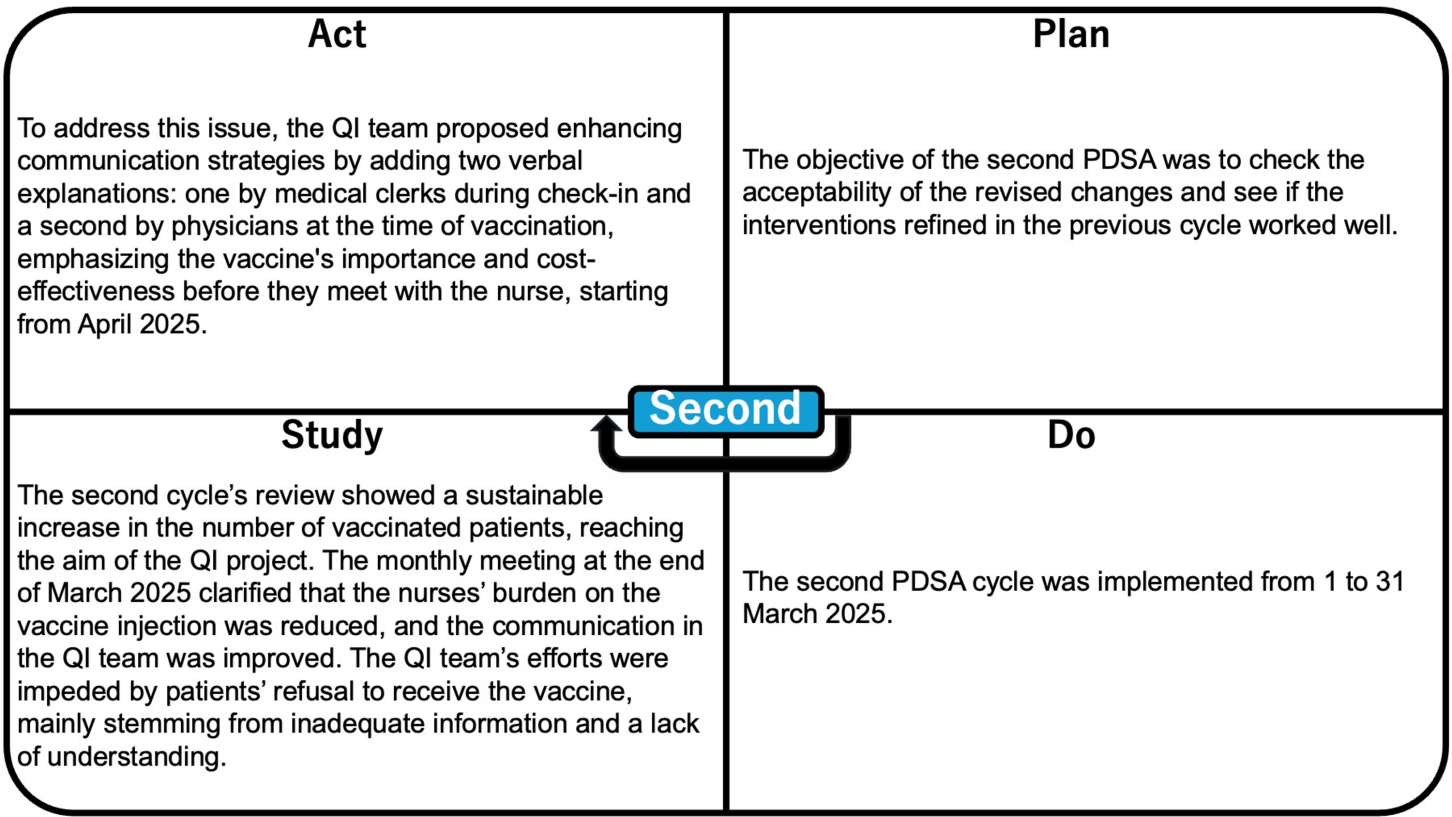
Process of the second PDSA cycle PDSA, Plan-Do-Study-Act; QI, quality improvement

The implementation of the change packages was carried out in phased stages, allowing the team to monitor each change’s effectiveness through sequential PDSA cycles. The processes are summarized in Figure 5 and Figure 6.

The first cycle was focused on my outpatient section, and the following cycle broadened the other family physicians’ sections.

Analysis

As part of the process measures, weekly rates for vaccination, recommendation, and reminders were calculated using the data recorded by medical clerks. The rates were expressed as percentages of eligible patients each week. For example, the vaccination rate was calculated as the number of patients vaccinated divided by the number of eligible patients, expressed as a percentage by multiplying by 100. These data were compiled and reviewed by the QI team. Run charts were created to visualize monthly trends in vaccination, recommendation, and reminder rates over time. This will highlight any upward trends following the implementation of each change.

We also performed an interrupted time series analysis (ITSA) to evaluate the impact of a change on pneumococcal vaccination rates among individuals aged 65 and older. Using a segmented regression model, we assessed baseline trends, immediate changes in vaccination rates after the change, and shifts in post-change trends. The model included terms for time, change, and the interaction between time and change. Diagnostic tests, including residual analysis and autocorrelation checks, ensured model validity. Sensitivity analyses evaluated the robustness of the findings by varying the time frames. Results highlighted the effectiveness of the change and informed future strategies.

Regarding satisfaction scores, the average ratings for each item on the six-point questionnaire were calculated at two times: after the first and second change cycles. These scores were used to monitor staff perceptions of the project’s effectiveness, workload burden, and workflow integration, providing a measure of acceptability and engagement over time.

Qualitative data from open-ended survey responses were analyzed using an inductive thematic analysis approach. The first author (RO) independently conducted line-by-line coding of all responses, generating initial codes through a process of constant comparison. Codes were then grouped into higher-order categories, which were refined into final themes through iterative discussion with the second and third authors (KK and CS). Analyst agreement was reached through consensus meetings. Triangulation was ensured by comparing codes derived from physicians, nurses, and medical clerks to capture multiple perspectives within the outpatient workflow. An audit trail was maintained to document coding decisions, enhancing transparency.

All statistical analyses were performed using EZR version 1.51 (Saitama Medical Center, Jichi Medical University, Saitama, Japan; URL: http://www.jichi.ac.jp/saitama-sct/SaitamaHP.files/OSXEN.html), a graphical user interface for R (The R Foundation, Vienna, Austria) [20].

Patient and Public Involvement

Patients provided feedback on the clarity and understanding of the brochure during informal consultations, which informed iterative improvements in the educational materials. However, they were not directly involved in study design or analysis.

Ethical considerations

Ethics committee approval was obtained from the Unnan City Hospital. This QI project was approved by the Unnan City Hospital Ethical Committee (approval code: 20240005).

## Results

Baseline data

A total of 160 patients were included in this QI project by reviewing the EMR of the hospital. The mean age was 77.8 (SD = 7.58), and the male rate was 46.2%. Based on the review of EMR from June 2024 to January 2025, the rate of vaccinated patients was 17.4% on average over the eight months (Table 2).

**Table 2:** Baseline rates of the participants who were vaccinated with the pneumococcal vaccine The numerator was 160.

Parameter	June 2024	July 2024	August 2024	September 2024	October 2024	November 2024	December 2024	January 2025
Number of patients who were vaccinated with the pneumococcal vaccination	28	28	28	27	27	28	28	29
Total vaccination rate	17.50%	17.50%	17.50%	16.90%	16.90%	17.50%	17.50%	18.10%

Outcome measure

Change in the QI Project

**Figure 7:**
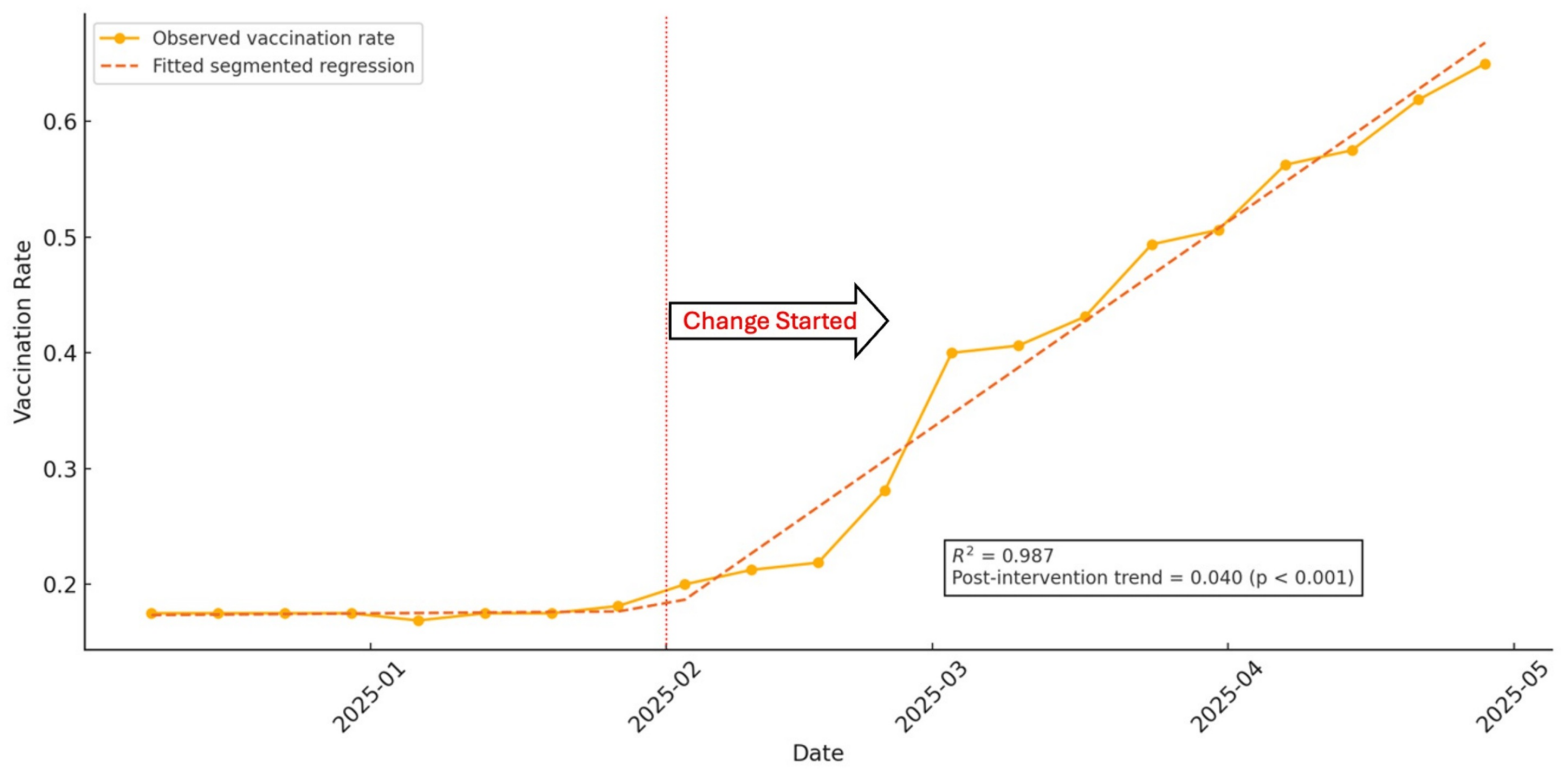
Run chart of pneumococcal vaccination rate and visual display of the ITSA The figure shows weekly pneumococcal vaccination rates among patients aged 65 and over during the QI project. The dotted line represents the fitted segmented regression. According to run chart rules [21], a sustained shift was observed, with more than six consecutive data points above the baseline median following the change, indicating a fundamental change in the system. No evidence of astronomical data points or prolonged trends was detected in the pre-change phase. A sustained shift above the baseline median and divergence of post-intervention slope illustrate a temporal change consistent with QI effects; however, the lack of a control period limits causal attribution. ITSA, interrupted time-series analysis; QI, quality improvement

During the three-month QI period, the rate of vaccinated patients increased from 17.4% on average before the QI project to 65.0% in April 2025. The number of patients who undertook vaccination increased from 27.8 patients on average before the QI project to 104 patients on April 28, 2025, resulting in improved vaccination conditions (Figure 7).

Analysis Based on the ITSA

The result of ITSA’s statistics is shown in Table 3.

**Table 3:** Statistical data of interrupted time series analysis

Parameter	Coefficient	Standard error	t-Value	p-Value	95% CI lower	95% CI upper
Const	0.1734	0.0141	12.3162	<0. 001	0.1437	0.2031
Time	0.0004	0.0034	0.1326	0.8961	-0.0067	0.0075
Intervention	-0.0299	0.0191	-1.5707	0.1347	-0.0701	0.0103
Post time	0.0397	0.0037	10.6207	<0. 001	0.0318	0.0475

ITSA showed no significant baseline trend (0.04 percentage points/week; p = 0.896; 95% CI: -0.0067 to 0.0075). After the intervention, the weekly trend increased significantly by 3.97 percentage points (p < 0.001; 95% CI: 0.0318 to 0.0475) (Figure 7).

Process measure

Brochure Supply and Demand

**Figure 8:**
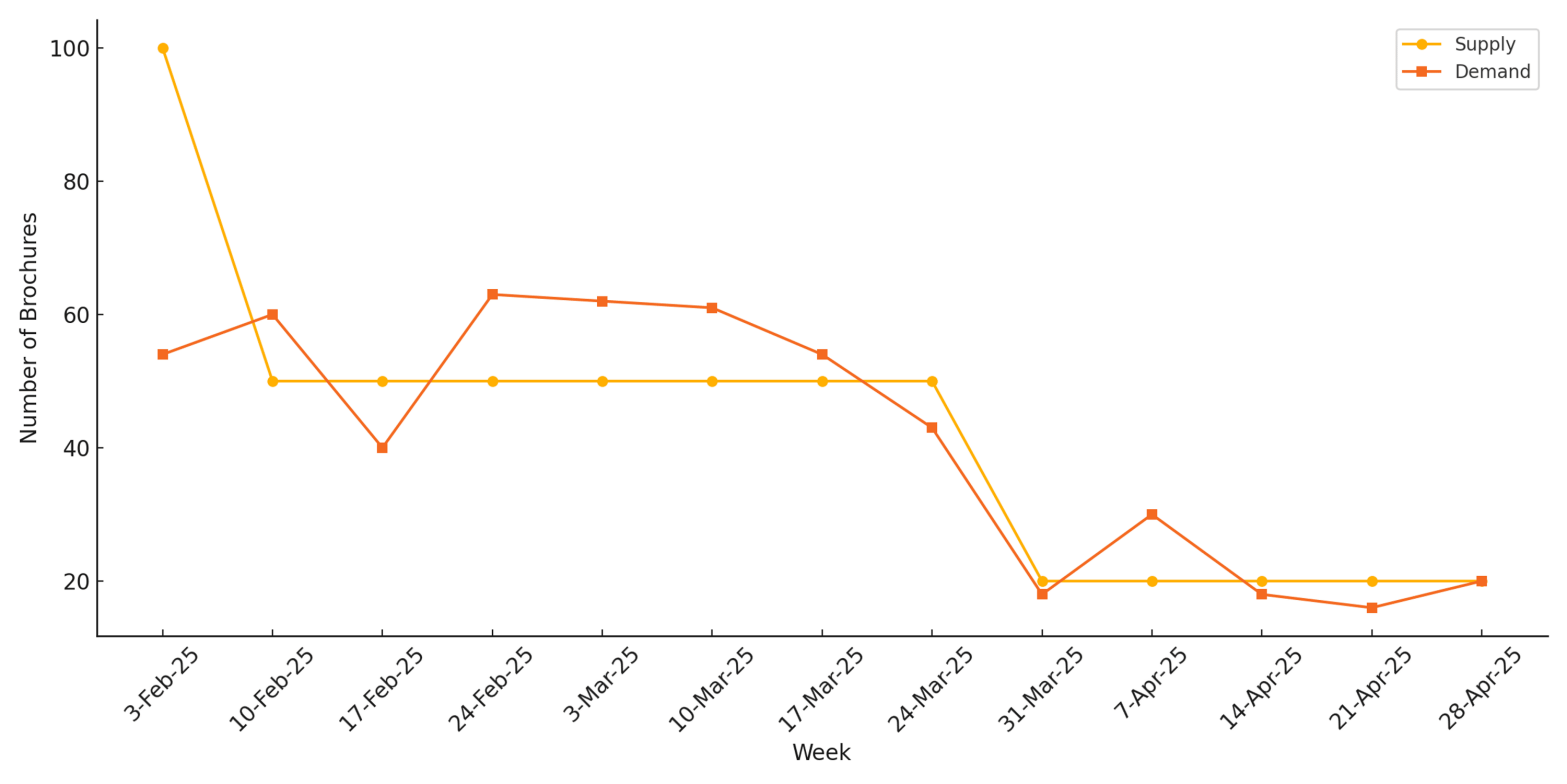
Weekly brochure supply and demand

Brochure demand and supply remained generally aligned across the observed period. Family physicians ensured that all patients received brochures after their consultations. Despite fluctuations in weekly demand, restocking rates consistently matched the demand, maintaining 100% coverage for vaccination-related information needs. This indicates the effective coordination between brochure provision and clinical consultations (Figure 8).

Vaccination Recommendation and Reminder

**Figure 9:**
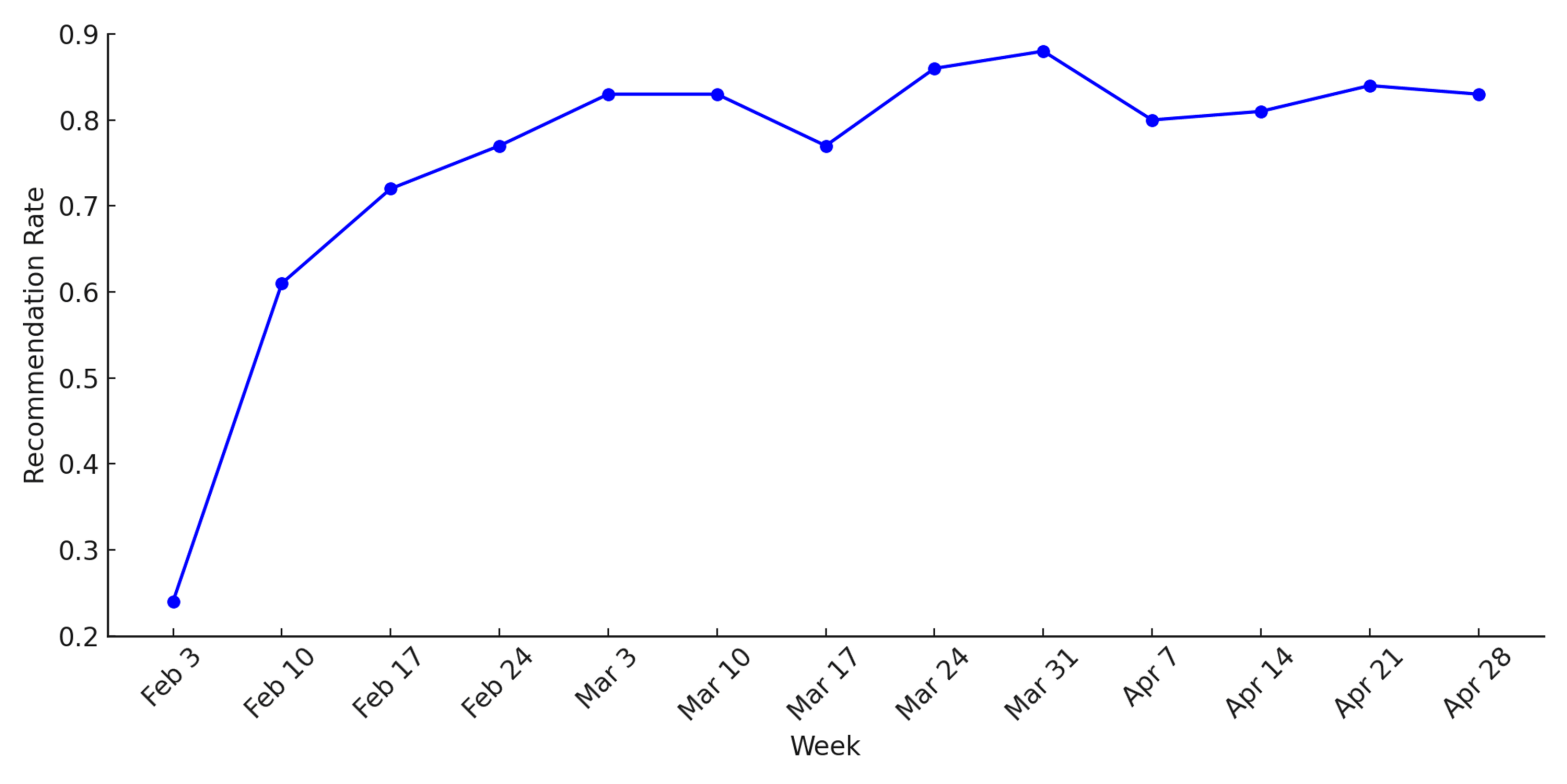
Recommendation rate over time

Physicians’ recommendation rates for vaccination drastically increased from 24% at the beginning of the QI project (early February) to an average of 83% in April 2025. This upward trend suggests effective implementation of physician-led recommendations in clinical practice throughout the post-change period (Figure 9).

**Figure 10:**
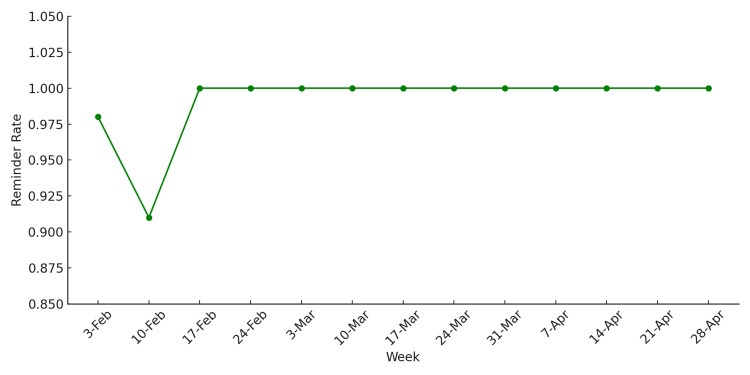
Weekly reminder rate during the QI project QI, quality improvement

The weekly reminder rate ranged from 91% to 100% during the QI project, with the rate reaching 100% consistently after mid-February. This demonstrates that the reminder system was well-integrated into the consultation process and sustained effectively throughout the post-change period (Figure 10).

Balance measure

QI Questionnaire Comparison

**Figure 11:**
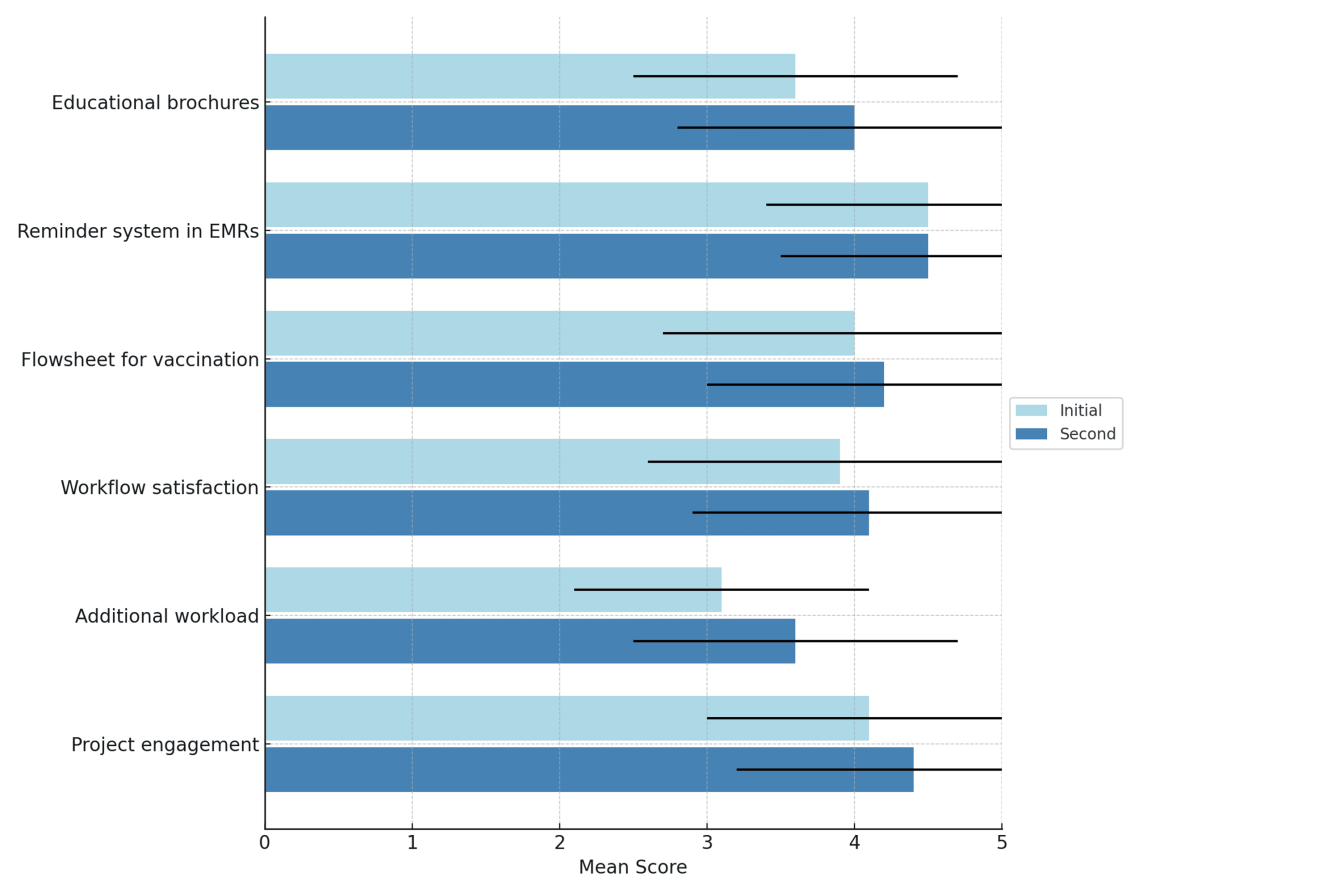
Comparison of questionnaire results before and two months later after QI changes QI, quality improvement

A comparison of the initial and second questionnaire results showed improvements across most categories. Project engagement increased from 4.1 (SD = 1.1) to 4.4 (SD = 1.2). Workflow satisfaction improved from 3.9 (SD = 1.3) to 4.1 (SD = 1.2). Educational brochures saw a rise from 3.6 (SD = 1.1) to 4.0 (SD = 1.2). The flowsheet for vaccination improved slightly from 4.0 (SD = 1.3) to 4.2 (SD = 1.2). The reminder system in EMRs remained stable at 4.5, with a minor improvement in SD (from 1.1 to 1.0). Additional workload, while still the lowest-rated item, improved from 3.1 (SD = 1.0) to 3.6 (SD = 1.1), suggesting increased acceptance of tasks related to the changes (Figure 11).

Qualitative Analysis of Open-Ended Responses

Thematic analysis of open-ended responses identified three major themes: (1) limitations in daily vaccination capacity; (2) communication challenges among professionals; and (3) patient misunderstandings regarding vaccination. Illustrative quotations have been added for transparency.

Theme 1: Limitation of daily vaccination capacity: Nurses described difficulty balancing routine clinical duties with increased vaccination demand. One nurse noted, “I hoped to vaccinate more patients, but my other tasks filled the entire shift.” This prompted the decision to cap daily vaccinations at five during the second cycle.

Theme 2: Communication challenges among professionals: Professionals expressed frustration with the misalignment of roles and the need for real-time coordination. A medical clerk reported, “Nurses were so busy that they didn’t hear me, and the patient became irritated.” This highlighted the need for improved intra-team communication.

Theme 3: Patient misunderstanding of immunization: Misconceptions about vaccine cost and necessity caused tension during consultations. One clerk shared, “A patient became angry because they misunderstood the cost and thought we were charging incorrectly.” This led physicians to strengthen risk-benefit and cost explanations during visits.

These insights informed iterative adjustments in the change process, as summarized with corresponding actions in Table 4.

**Table 4:** Themes identified and corresponding actions

Theme	Description	Representative quote	Action taken
Limitations of daily vaccination	Nurses struggled to balance vaccination with other duties.	“I hoped to vaccinate more patients, but I was full of other work…”	Daily vaccination was limited to five patients from the second cycle.
Lack of communication among professionals	Miscommunication hindered collaboration and upset patients.	“Nurses become busy and do not listen to me… patients were irritated.”	Communication about patient perceptions was enhanced among the care team.
Misunderstandings about immunization	Patients misunderstood vaccine costs and benefits, leading to frustration.	“They did not understand the cost of vaccination and got angry with me.”	Physicians improved their explanations, including vaccine risks and associated costs.

## Discussion

Summary

This QI project successfully enhanced pneumococcal vaccination uptake among older patients in a rural Japanese hospital by implementing a multicomponent change, including educational brochures, EMR-based reminder systems, and a streamlined vaccination flowsheet. The vaccination rate improved from an average of 17.4% before the QI project to 65.0% over a three-month QI period. The initiative was supported by consistent physician engagement, improved workflow coordination, and high satisfaction scores from participating staff. Although the increase from 17.4% to 65.0% is substantial, this improvement should be interpreted cautiously. The short time frame of the QI project, high team motivation, and heightened awareness among staff may have amplified performance, consistent with a Hawthorne effect. These contextual influences were not measured quantitatively and likely contributed to the rapid increase observed. Therefore, while the findings demonstrate a strong temporal association, they do not establish causal generalizability beyond this specific setting.

Comparison with other studies

These findings are consistent with existing literature demonstrating that provider-targeted and system-level changes significantly improve adult immunization rates. A meta-analysis including changes such as computerized reminders and patient-directed education reported an overall pooled odds ratio of 4.33 (95% CI: 2.02-9.39) for increased vaccination uptake [16]. Similar to Zimmerman et al. (2017) and Ho et al. (2019), our results suggest that integrating low-cost educational and reminder strategies into outpatient practice is both feasible and impactful, even in resource-limited rural settings [12,14].

Strong points of this study

Several features contributed to the success of this initiative. First, the design change was grounded in evidence and tailored for rural healthcare settings, aligning with the adaptation frameworks described by Castro et al. (2010), who emphasized the importance of culturally and contextually appropriate implementation [22]. While their work focused on adapting behavioral changes, our project applied similar principles by selecting low-cost, scalable tools such as brochures and EMR-based reminders that fit a rural outpatient clinic’s workflow and resource availability.

Second, the approach emphasized interdisciplinary engagement, promoting shared responsibilities among physicians, nurses, and clerical staff. This collaborative model reflects the strategies described by Will et al. (2019), who demonstrated that interprofessional team structures lead to improved implementation of preventive changes [23]. However, while their study took place in larger urban healthcare systems, our project adapted this model to a smaller-scale, rural setting by integrating clerks more actively in patient education and reminder systems.

Third, the project incorporated real-time feedback through questionnaires and team discussions, which supported high engagement. Like Will et al.’s findings on the role of adaptive leadership and feedback loops, our team responded to concerns about daily workload by limiting vaccinations to five per day, an example of flexible, context-driven adjustment to maintain staff motivation and workflow stability.

Limitations of this study

Despite its strengths, the project has limitations. The study was conducted in a single rural hospital, which may limit its generalizability to other institutions, especially those with different organizational structures or resource availability. The short duration of the evaluation also limits our understanding of the long-term sustainability of the observed improvements. Given the rural outpatient context, small team structure, and close collaboration among physicians, nurses, and clerks, the external validity of our findings is inherently limited. Similar results may not be replicated in larger institutions with more complex workflows or different EMR systems. The intervention components were designed for a low-resource rural environment, and their effectiveness may vary across healthcare systems.

Additionally, while staff satisfaction and process measures were assessed, patient satisfaction and broader outcome measures (e.g., hospitalization rates or incidence of vaccine-preventable pneumonia) were not included. Finally, the possibility of a Hawthorne effect, where individuals alter their behavior due to awareness of being observed, cannot be ruled out. Although ITSA strengthens the evaluation of temporal changes, the study did not include a contemporaneous control group or external comparator. As a result, unmeasured factors occurring during the project period cannot be definitively excluded. Therefore, the findings reflect associations rather than causal effects, and the observed improvements should be interpreted within the inherent constraints of an uncontrolled QI design. 

The way forward

Looking ahead, the change framework developed here may be expanded to other healthcare settings, including small clinics and other rural hospitals. For successful adaptation, key contextual factors include having a small, consistent outpatient team with close communication between physicians, nurses, and clerical staff. A basic EMR system that allows for customizable reminders is also essential. Additionally, settings that value team-based care and encourage feedback-driven QI are more likely to benefit. Cultural factors, such as patients’ trust in healthcare providers and local norms around preventive care, should also be considered to ensure effective communication and engagement.

Beyond short-term gains, sustainability is essential for meaningful improvement. To support long-term durability, the QI team has initiated several sustainability mechanisms: (1) embedding the EMR-based reminder into routine outpatient templates; (2) establishing a standing policy that requires vaccine eligibility checks during chronic disease follow-up visits; (3) integrating the flowsheet into staff onboarding materials; and (4) initiating quarterly performance monitoring to track vaccination rates and identify drift. These institutional supports aim to maintain improvements even after initial enthusiasm wanes, addressing a common challenge in time-limited QI projects.

Ultimately, the effectiveness of this intervention should be judged not only by immediate improvements but also by the extent to which the processes can be sustained and adapted over time. Embedding the workflow changes into routine practice and maintaining periodic monitoring will be critical to ensuring long-term impact.

## Conclusions

This QI project demonstrated that simple, low-cost, workflow-integrated strategies, such as EMR-based reminders, educational brochures, and standardized vaccination flowsheets, were associated with substantial increases in pneumococcal vaccination uptake among older adults in a rural outpatient setting. However, because the project lacked a control group, causality cannot be confirmed, and the observed improvement should be interpreted as a temporal association rather than a definitive effect of the intervention. To support scalability across other rural systems, the intervention required minimal resources: brochure printing, brief EMR modifications by in-house IT staff, and approximately two to three hours of training for nurses and clerks. The staff-to-patient ratio enabled the integration of reminders without requiring additional personnel, suggesting feasibility in similar low-resource settings. Embedding reminders into routine workflows and quarterly monitoring may support long-term sustainability and successful spread to other rural clinics and hospitals.
